# Measuring the Impact of Armed Conflict on Population Health: A Guide for Researchers

**DOI:** 10.1111/padr.70047

**Published:** 2026-03-11

**Authors:** Maya Luetke, Signe Svallfors, Elizabeth Heger Boyle

**Affiliations:** Minnesota Population Center, University of Minnesota, Minneapolis, MN 55455, USA.; Department of Sociology, Stockholm University, 10691 Stockholm, Sweden.; Department of Sociology, University of Minnesota, Minneapolis, MN 55455, USA.

## Abstract

The humanitarian impact of armed conflict remains a significant international issue, with an estimated 2 billion people residing in fragile or conflict-affected settings. Despite increasing attention and study of armed conflict and its impact on human populations, few studies have evaluated the methods necessary to assess such relationships: how to use disaggregated and granular conflict data, measure and operationalize conflict, etc. In this study, we identify important considerations for conducting armed conflict and health research, including how data structures and decisions might impact conclusions. We discuss the particular characteristics of existing armed conflict datasets and the types of biases that may be present in data drawn from conflict-affected areas. Further, we demonstrate how data and measurement choices can result in different conclusions and, if handled improperly, even spatial misclassification, bias, and spurious conclusions. Lastly, we illustrate some of these data and measurement choices in an empirical example where we assess the impact of conflict on women’s contraceptive use in Nigeria. Using conflict event data from the Uppsala Conflict Data Program and health data from the Nigerian Demographic and Health Survey in 2018, we show how the relationship between exposure and outcomes varies across different spatiotemporal dimensions of conflict exposure measurement.

## Introduction

Armed conflict is a substantial threat to health and well-being, contributing to increased mortality and morbidity, destruction of social and physical infrastructure, health service disruption, disruptions of agricultural activities and livelihoods, and mass displacement, among other adverse impacts (Akil and Ahmad 2016; Bendavid et al. 2021; Echevarria-Coco and Gardeazabal 2021; Garry and Checchi 2020; Levy and Sidel 2016; Wise 2017). Over the short- and long-term, conflict may lead to food, water, and other resource scarcities (Brück and d’Errico 2019; Martin-Shields and Stojetz 2019) as well as increased transmission of infectious diseases (Wise and Barry 2017; Raad et al. 2018; Akil and Ahmad 2016). Although men are generally more likely to be direct victims of conflict violence due to their overrepresentation as combatants, women and children are often disproportionately harmed by the indirect consequences of conflict because of disadvantage caused by persistent gender-based social and power inequities (Bendavid et al. 2021; Garry and Checchi 2020; Kadir, Shenoda, and Goldhagen 2019). The impacts of armed conflict are often felt widely through communities and may hinder or even reverse progress on development goals, thereby further intensifying poverty among affected populations (Samman et al. 2018).

Given the widespread social, economic, and health impacts of armed conflict, researchers are increasingly studying these relationships. With recent methodological and data advancements, researchers can now use sophisticated quantitative analyses that empirically measure and assess the impact of violence on population health at the individual level (Wise 2017). By leveraging spatiotemporal variations in conflict exposure in this way, it is possible to employ quasi-experimental and causal inference techniques that help distinguish specific effects of violence from differential propensities of being exposed *and* enduring poor health outcomes. These methodological advancements have created new opportunities for systematically studying the impact of war on population health, which has sparked a substantial and growing literature in this field.

However, research to date lacks consistent definitions, measurements, and processes for evaluating armed conflict exposure. While there are some common conventions in the measurement of armed conflict exposure, like using conflict event or fatality counts, there are no standards for measuring exposure to conflict even when researchers use the same datasets. There is a need for a comprehensive, systematic, and data-informed evaluation of how to operationalize exposure when researching population health and armed conflict, including how data structures and model decisions might impact conclusions.

To address this gap in the existing evidence base and to guide future research in this field, we offer an overview of important considerations for conducting research on population health in armed conflict, including how data structures and decisions might impact conclusions. In this study, we address a few main considerations including (1) identifying, and dealing with, imprecision in armed conflict location data and health data; (2) deciding on appropriate spatial and temporal aggregation of conflict and health data to measure exposure, (3) operationalizing conflict exposures, and (4) using appropriate analysis methods to answer the specific research question. We also cover several other important aspects of conducting armed conflict research, such as understanding possible sources of bias. Lastly, we provide an empirical example of how to measure, aggregate, spatially join, and conduct an analysis of the effect of armed conflict on population health, in this case, current contraceptive use in Nigeria. While this methodological study is focused specifically on linking armed conflict and health data, the approach described here will likely also apply to other conflict-related research that measures individual-level exposure to conflict and seeks to match armed conflict data with other data sources. Our study contributes to discussions aiming to improve methodological approaches with the goal of yielding rigorous, accurate, and unbiased study results. Our empirical example also adds to the current literature on the effects of armed conflict on reproductive health outcomes (Luetke et al. 2024; O’Brien 2020; Svallfors and Billingsley 2019; Torrisi 2024; Williams et al. 2012; Thiede et al. 2020; Oskorouchi 2019; Østby et al. 2018; Torrisi 2023; Svallfors et al. 2024a).

### Research process: Armed conflict and individual health

The appropriate research strategy and process for studying the relationship between armed conflict and health outcomes will differ for each research question and study, thus requiring study-specific data preparation, measurement of conflict exposures, and operationalization of said exposures. Such decisions should be explicitly and transparently explained in the methods section of papers. However, many studies lack transparency in such decisions, overlooking or providing overly simplified descriptions, with key details omitted. This opacity makes it difficult both to assess the validity of the variable constructs, to compare models and findings across studies and to replicate the results. Describing how exposure to conflict was measured is critical to advancing the field of armed conflict and health, particularly by allowing for comparison between studies and innovation in methods.

The research process for conducting quantitative research on armed conflict and individual health typically starts with identifying appropriate sources of georeferenced armed conflict data and health data. In the first four subsections below, we discuss the specifics of the most popular armed conflict data and health data. Next, we explain some of the most important decisions that must be made when linking armed conflict data to individual-level health data.

#### Armed conflict datasets

The existence of subnational data on armed conflict events has transformed research on armed conflict, which formerly relied solely on gross country-year measures (Eck 2012). Out of many existing conflict datasets, the two most commonly employed to monitor and research armed conflict are the Uppsala Conflict Data Program Georeferenced Event Dataset (UCDP-GED) (Croicu and Sundberg 2016) and the Armed Conflict Location and Event Data (ACLED) (Raleigh et al. 2010). While national datasets may be more relevant in some cases, these global datasets enable comparisons across contexts and have been widely used in population health research. Although their approaches for finding information vary somewhat, both UCDP-GED and ACLED rely primarily on news stories and reports from nongovernmental and humanitarian organizations to determine the details of conflicts, including where and when they occurred, who was involved, and the number of fatalities, if any.

The ACLED and UCDP-GED datasets are not equivalent because of differences in how conflict is defined and operationalized (see Table 1). ACLED gathers information on “violent events,” defined as single altercations involving the use of force for political ends. Within ACLED, fatalities are not a prerequisite for something to be counted as a violent event. UCDP-GED, in contrast, records “ongoing political conflicts,” which typically include a *series* of violent events. UCDP sets a threshold of 25 fatalities within a calendar year for an engagement to be considered an ongoing political conflict; in addition, each event within the series must be associated with at least one fatality (Sundberg and Melander 2013; Croicu and Sundberg 2016). ACLED aims to record not only large-scale, well-publicized events but also smaller, less-publicized armed conflicts. Because of the differences in measurement, ACLED typically reports more conflicts than UCDP-GED (Dawkins 2021; 2025). Both datasets are potentially valuable, but they are not interchangeable.

UCDP-GED provides the start and end dates of each armed conflict event included in the dataset, the geolocation of these events, and additional details such as the actors involved in the conflict and estimates of the number of fatalities (Croicu and Sundberg 2016). The dataset also includes indicators of temporal and spatial precision, reflecting the level of certainty or accuracy of the reported timing and location of the armed conflict event. For example, these indicators specify whether the precise location is known or only a broader spatial area, such as a region, and whether the exact day or month is reported or only a less specific temporal range, such as the year. Armed conflict events may sometimes span multiple days, weeks, or even longer (ACLED 2024; Croicu and Sundberg 2016). In the UCDP-GED dataset, such multiday events are typically represented as a single observation, with both a start and end date provided to indicate the duration of the armed conflict event.

By comparison, ACLED records the type of event (i.e., the form of violence), the number of fatalities (if available), the source of the report, and temporal and spatial details of the event. Like UCDP-GED, ACLED includes indicators of spatial and temporal precision. For example, it distinguishes between events where the exact location is known and those where the location is extrapolated to a broader administrative unit (e.g., district capital), due to a lack of information about where the armed conflict event occurred (ACLED 2024). A key difference between UCDP-GED and ACLED is that ACLED represents each day of an ongoing conflict event as a separate observation in the dataset. ACLED handles events in time slightly differently, with each day of an ongoing armed conflict event being represented by a new observation in the dataset. Nevertheless, ACLED does also include a temporal precision variable indicating the confidence in the reported timing of the event.

UCDP is generally considered a more conservative measure of armed conflict with a specific focus on lethal violence due to its fatality quota for reporting. Given its longer historical coverage and annual updates, it is often preferred for cross-national or longitudinal analyses of trends in armed conflict as well as for studies that are specifically focused on lethal violence. In contrast, ACLED’s near real-time reporting and more inclusive definition of violence make it particularly useful for tracking ongoing and active conflicts as well as analyzing a broader set of violent events—including non-fatal violent events such as protests and riots that may not meet the fatality threshold of UCDP inclusion. For example, violent episodes and events can have significant social and health impacts regardless of whether they result in fatalities.

Although UCDP and ACLED are among the most widely used armed conflict datasets, several others may also be relevant for research and are worth mentioning here. These include the Global Terrorism Database; the Integrated Crisis Early Warning System dataset; the Phoenix event dataset; and the Global Database of Events, Language, and Tone (Raleigh and Kishi 2019).

#### Potential sources of bias in armed conflict datasets

Collecting reliable and accurate conflict event data often poses significant challenges, and researchers should be aware of the potential gaps, errors, and biases in these data (Dawkins 2021; 2025; Chojnacki et al. 2012; Von Borzyskowski and Wahman 2021). In ongoing conflict, agencies and actors that document and report armed conflicts may have various motivations for purposefully under- or over-reporting armed conflict events and may differ in their ability (i.e., technical skill) to assess, investigate, and determine whether or not a conflict event has occurred. As a result, data on armed conflict events—especially from active or remote conflict zones—may be incomplete or inaccurate. Key sources of potential bias include underreporting, potential spatial gaps in coverage, and biases introduced by reliance on secondary reporting or limited ability to verify conflict events happened in active conflict zones. Accordingly, it is both important to understand and discuss the limitations of these data when using them for research and analysis.

##### Reporting and recall bias.

During active or ongoing conflicts, armed groups and other involved parties may actively work to conceal their violent acts or otherwise mislead investigators. Because information can be dangerous to collect and misinformation (or control of narratives) is often weaponized in armed conflict, with various sides having motivations for spreading false information, there can be significant inconsistencies in the reporting of specific conflict events as well as where they occurred. For example, groups involved in a particular conflict may have strategic interests in obscuring information about combatant movements and conflict event locations. They may not want to reveal losses (i.e., casualties) to portray strength or want to obscure the location of a particular conflict event so that combatant locations remain unknown to the opposing side and cannot be targeted.

Some suggest counting fatalities rather than events eliminates more of this bias because fatalities are more difficult to conceal (Eck 2012), which would imply that the UCDP-GED may be more useful. However, others suggest that fatality reports may be particularly biased and unreliable (Raleigh, Kishi, and Linke 2023). Dawkins (2021) determined that both ACLED and UCDP tend to significantly underreport fatalities and recommends that researchers utilize strategies to reduce such bias, including using categorical rather than continuous measures of fatalities. Though not a panacea, categorical transformations tend to reduce sensitivity to small reporting errors, as the reported event with a reporting error in fatalities may still fall into the same category it would if the fatalities had been reported accurately. Additionally, using a categorical variable instead of a continuous measure reduces spurious associations as it reduces the influence of extreme values and outliers. However, it is a cruder measure when compared to more exact, continuous data and also presents new problems, such as what thresholds represent meaningful levels of violence (e.g., high versus low violence).

Other parties involved in the reporting and recording of armed conflict, such as journalists and news outlets, may also have motivations that influence the reports of conflict events and the resultant datasets. Both news outlets and journalists have economic interests and, therefore, may be influenced to publish narratives that get readership. This may involve prioritizing the reporting of armed conflicts that are perceived to have more international significance, or that play into dominant, but not necessarily accurate, narratives (e.g., that conflict in poor countries is driven by ethnic animosities) (Whipple and Shermak 2018). Additionally, the technical skill and expertise of reporters or investigators on the ground (e.g., forensic scientists, journalists, or volunteers) can greatly affect discovery and reporting of conflict events. Even when the parties involved in reporting conflicts have the best motivations and skills, some biases are still likely to occur. Areas that are more accessible (e.g., urban or with cell phone coverage) tend to have better reporting, while remote, harder-to-access areas may have no coverage at all.

##### Imprecision.

Reports of armed conflict events may lack key details, such as precise geographic or temporal indicators. They often refer to large areas, such as provinces or territories, or their descriptions of locations contain ambiguity, such as “Tingi-Tingi refugee camp (130 km southeast of Kisangani)”, “Kikwit location (400 km (250 miles) east of Kinshasa and around the town of Kenge)”, or “Bekeli area (three villages near Bukungu, in Bekeli, Nkembe, Bekili, and Zongo settlements)” (Uppsala Conflict Data Program (UCDP) 2023). The conflict dataset coders must use this general information to identify the latitude and longitude of conflict events, which then introduces measurement error into the data. Further, conflict data in rural areas may be more under-reported and less precise than in more populated areas due to differential reporting patterns. This issue may be particularly salient in rural, long-term conflicts, in which geographic marginalization might affect data availability and quality.

A lack of precise spatial and temporal details can present significant challenges to linking other datasets (see Table 2). Because conflict datasets are limited in their ability to accurately obtain and report when and where certain events occurred, some records have an exact location, while others only have a broader estimation or approximation of the location where the event occurred. For events lacking an exact location, UCDP-GED and ACLED each have a defined protocol. These differ slightly but involve aggregating to a broader spatial level, sometimes the centroid of an administrative subdivision or a nearby town or city (ACLED 2024; Croicu and Sundberg 2016; Sundberg and Melander 2013). However, this data feature is often overlooked by researchers, going either completely unaddressed or without a transparent description of how it is used. A lack of appropriately addressing issues such as the imprecision of some of the reported conflict event locations may lead to spatial misclassification and possibly spurious associations and/or biased estimates.

Precisely how the potential biases will affect conflict-and-health analyses will vary, so researchers need to independently assess their most effective approach, depending on the study context and research questions. Further, in any resulting publications, researchers should provide an explicit and transparent account of the inherent and particular limitations of their analysis, including the possible biases and the plausible directional impacts of said bias, in any resulting publications.

#### Population health datasets

Population-representative health surveys—such as the Demographic Health Surveys (DHS; dhsprogram.com), Performance Monitoring for Action (PMA; pmadata.org), and Multiple Indicator Surveys (MICS; mics.unicef.org)—often provide information about the location of data collection. Other commonly used population-representative health surveys that may be useful include: The Living Standards Measurement Study, Service Provision Assessment surveys, Malaria Indicator Surveys, among others, and more country-specific surveys. Some surveys provide Global Positioning System (GPS) coordinates, which are usually displaced or offset to protect the identity and privacy of survey respondents. There are multiple ways to address GPS displacement when using population health datasets (Boyle et al. 2020; Grace et al. 2019; Perez-Heydrich et al. 2016). When using DHS, it is recommended to employ buffers ranging from 1 to 5 km for urban clusters and 1–20 km for rural clusters (Boyle et al. 2020; Perez-Heydrich et al. 2016). Researchers may want to consult other resources before deciding what buffer sizes to employ and consider displacement protocols of each specific survey, as well as the possible distance at which nearby violence may have an impact on their particular analysis (Boyle et al. 2020; Grace et al. 2019; Perez-Heydrich et al. 2016). In other words, the choice of buffer size should be informed by contextual knowledge of the particular conflict and setting of the study as well as the nature of the research question. Researchers may also want to conduct sensitivity analyses across a range of different buffer areas (i.e., localized armed conflict inclusion areas) to assess whether the findings are robust and similar across various buffer sizes.

In cases where geocodes are lacking, many surveys contain information about the administrative boundary levels 1 and 2 of the respondents’ residence. In such cases, the researcher needs to accurately match the corresponding values in locations since the names of geographic subdivisions can vary in their spellings and sometimes may even be used multiple times within the same country. One way is to use unique alphanumeric codes of administrative subdivisions provided by the International Organization for Standardization (in particular, ISO 3166–2). However, this can be a strenuous process depending on the number of administrative subdivisions in a particular geographic context. Another way is to use the unique combination of units at both level 1 and level 2 administrative subdivisions.

#### Potential sources of bias in population health datasets

Collecting health data during conflict is complicated, sometimes even impossible, due to restricted movement or displacement, limited communications, and acute threats to civilian lives and safety. Therefore, health microdata from or near conflict zones may lack accuracy and reliability, which requires caution and an explicit discussion of data limitations. Some of the most important potential sources of bias include sampling, selection, reporting, and recall biases.

##### Sampling bias.

Limited access to conflict zones, logistical constraints, and security concerns can influence the ability to sample respondents in highly conflict-affected areas. For example, respondents may be forcibly displaced or conscripted to join armed groups. Lack of access to conflict-impacted communities may lead to sampling bias and reduce the overall response rates, leading to a lack of representativeness—even in datasets aiming to ensure a representative sample—and an incomplete knowledge about the experience, scope, and impact of armed conflict. While data are often difficult to collect during active conflict, there are some common ways to address this issue, including employing retrospective data collection modules that ask respondents to recount past health outcomes and experiences that may have overlapped with the more severe or intense periods of armed conflict. With such data, the analyst would be able to create a quasi-longitudinal dataset, providing health information that temporally corresponds to periods when conflict may have been too severe or intense to permit simultaneous or real-time data collection.

For some health surveys, such as the DHS, such challenges are typically identified in the final reports. In general, reading such reports is important to understand the specific idiosyncrasies of data collection and structure. When surveys are administered during active conflict, the DHS Final Report often includes a statement specifying which districts were excluded from the sample for security concerns. For example, the DRC 2013–2014 Final Report states: “During the EDS-RDC II, a sample of 540 clusters was drawn, of which 536 could be visited. Four clusters (two in Katanga, one in Province Orientale, and one in North Kivu) could not be visited due to insecurity” (Ministère du Plan et Suivi de la Mise en œuvre de la Révolution de la Modernité - MPSMRM/Congo, Ministère de la Santé Publique - MSP/Congo, and ICF International 2014, 13). Understanding the representativeness of a given dataset is an important consideration for any study, as it can have significant impacts on the conclusions.

##### Survival and selection bias.

Selection bias occurs when the sampled population includes only certain subpopulations. Selection bias resulting from mortality (i.e., survivorship bias) and population displacement may lead to inaccurate inferences about the effect of conflict on population health because individuals who have died or emigrated will be excluded from the study. Because men tend to be more likely to be involved as combatants in armed conflict, they may be more likely to be displaced, injured, or killed and, thus, unrepresented in survey data. Similarly, some amount of displacement usually occurs in response to armed conflicts (Echevarria-Coco and Gardeazabal 2021), and this movement may also result in people being excluded from the data. Most importantly, subpopulations that died or migrated may differ substantially from the overall population. For example, individuals with more resources may be able to evacuate insecure areas while more vulnerable people have no option but to remain. In general, large-scale surveys that claim to be population-representative do not account for displaced or migratory populations and, therefore, cannot be used to understand the health experiences of these populations.

Selection bias due to migration patterns or mortality trends may impact who is included in a particular sample for data collection, thus reducing the representativeness of the sample. If, for example, the most well-off individuals (e.g., more affluent, healthier, able-bodied) are able to migrate away during conflict, then we may overestimate the negative impacts of armed conflict among the remaining population because the sample excludes those who left (Checchi et al. 2022). In contrast, if armed conflict is more likely to displace (or kill) the most vulnerable individuals (e.g., impoverished, chronically ill, malnourished), then we might underestimate the negative impacts of armed conflict since the sample disproportionately includes survivors and those who did not migrate away (Checchi et al. 2022; Degomme and Guha-Sapir 2010).

However, despite the understanding that armed conflict may and often does displace populations, the impact of conflict on out-migration may not be as large or as straightforward as expected with the majority of populations staying put even through the highest intensity of conflicts or through the lower intensity conflict and moving only at a high level of localized violence (Bohra-Mishra and Massey 2011; Williams 2015; Williams, O’Brien, and Yao 2021). Further, migration is a complex process that is influenced by a variety of factors and, correspondingly, armed conflict has often been shown to have complicated, context-specific, and often nonlinear influences on migration trends (Bohra-Mishra and Massey 2011; Erdal, Mjelva, and Tollefsen 2025; Williams 2015).

Some ways to address selection bias include comparing census data on population shifts within areas before and after a conflict and/or measuring population movement. However, such information is not always available. For example, the DHS does not report migration histories and only sometimes includes an indicator for how long respondents have lived in the residence at the interview and where they lived before. Though not standard, some DHS rounds in some conflict-affected countries (e.g., Nepal 2016, Pakistan 2017–2018, Turkey 2018) have collected migration and displacement histories including whether conflict was the reason for displacement (Le Voir 2023).

If available, migration and time-in-residence indicators can be used to include or exclude respondents based on whether they were living in a particular area during the time of exposure measurement. This type of information is critical to ensure that the assignment of exposure (or lack thereof) to violence is accurate. However, it is also not a perfect solution as shorter moves and returns may not be disclosed. Further, many of these variables and exposures are time-varying and, under ideal circumstances, we would be able to evaluate the time-varying exposure to violence over time and moves/displacement. However, such fine-grained, longitudinal population-representative data that follows individuals across time and space does not yet exist. Additionally, migration and displacement are also time-varying and there are some new methods of assessing such patterns in near real time, such as mobile phone call detail records, social media and other digital trace data, and satellite imagery (e.g., looking at nighttime light pollution). However, many of these innovative data sources face significant challenges in protecting the identities and safety of individuals, ethical issues in the procurement of these data (as they are often passively collected), and in connecting individual migratory/displacement patterns with corresponding health data. Consequently, researchers may simply have to acknowledge these types of bias as inherent to the study of the effects of armed conflict.

##### Reporting bias.

Security and safety concerns during conflict may make individuals wary or unable to report on their experiences, and logistical or infrastructure limitations may limit outreach during data collection. Non-reporting and/or underreporting may produce notable data omissions, thereby preventing the encapsulation of the full experience of people affected by conflict. Distrust and misinformation can also be prevalent in times of armed conflict and may even be weaponized by armed actors (Erlich and Garner 2023; Lewandowsky et al. 2013).

Gauging survey response rates is an important element of assessing survey data quality. For instance, response rates during the fielding of the longitudinal Chitwan Valley Family Study amid civil war were consistently and surprisingly high, at >90 percent individual response rates during 1997–2007, although bombings in local areas depressed response rates, and nationwide political events increased response rates (Axinn, Ghimire, and Williams 2012). In Colombia, the DHS has run every fifth year since 1986, despite an internal armed conflict that has endured since the 1960s, with response rates >85 percent throughout. According to representatives of the implementing organization of the Colombian DHS, Profamilia, the timing of fieldwork was sensitive to conflict, as the organization coordinates with local authorities and NGOs to determine when it is safe enough for fieldworkers to visit a community for data collection. Male response rates, but not female, have likely been affected by war because men are more directly involved in conflict than are women. This is also a form of selection or survival bias, which we discussed in the previous subsection in more depth. To access remote areas across Colombia’s dynamic terrain, Profamilia sent fieldworkers on donkeys through the jungle and across the Andes, negotiating with non-state armed groups such as the Revolutionary Armed Forces of Colombia (FARC) to enter areas under their control. Since Profamilia is a trusted nonprofit, private organization well known for its service provision via mobile clinics throughout Colombia, their fieldworkers were allowed to enter areas where governmental health service organizations were not (personal communication with Signe Svallfors, February 2018).

##### Recall bias.

Recall bias is a well-documented phenomenon, particularly in cross-sectional survey data which asks respondents to recall events retrospectively (Beegle, Carletto, and Himelein 2012; Bell et al. 2019). For example, there are several modules in the DHS that ask respondents to recall contraceptive use and pregnancy timing several years prior. Some such events, like the birth of a child, may be easier to recall because of their heightened importance while remembering the timing of changes between contraceptive methods several years back may be much more difficult to recall (Pullum and Becker 2014; Schoumaker 2014). Recall bias may also be influenced by social characteristics and, potentially, cultural conceptualizations of chronological time (e.g., sequential or cyclical) (Tumlinson and Curtis 2021). Generally, the greater the distance between data collection and the timing of the event that is being queried the greater the risk of recall bias with events being misreported or their timing being misremembered (Bell et al. 2019). Recall bias in the timing of events is particularly problematic when the misdated events serve as the exposure or outcome in an analysis, as this can distort the temporal alignment between armed conflict and the variable of interest in order to appropriately align exposure and outcome for analysis, undermining accurate analysis and the possibility of causal inference. Further, a common limitation for conflict and health analyses is that health data cannot be collected in active conflict zones, thus often necessitating retrospective data collection and the possibility of recall bias.

#### Spatial and temporal aggregation of conflict data to assess exposure

Despite growing academic attention on the effect of armed conflict on population health, few studies have carefully outlined the reasoning, logic, and assumptions that underlie their operationalization of exposure to conflict. Accordingly, several important considerations are often overlooked in this field of research, including the varying degrees of spatial and temporal imprecision of these data.

##### How to address spatial imprecision.

To deal with the spatial imprecision innate to armed conflict datasets, described above, researchers must decide whether to exclude events that lack detailed spatial precision or to aggregate to a higher spatial level. Removing events that lack spatial precision may bias results if conflict actors have purposely obfuscated locations. While the number of events without spatial precision is small in some conflicts, in others it can be quite large. In Nigeria in 2018, for example, 46 percent of conflict events had a reported spatial precision of the exact location, 20 percent had the location reported to be within a 25-km range, and 27 percent of the events could only be linked to second-order division (known as local government areas, LGAs, in Nigeria) (see Figure 1 and Table 3). In this case, simply omitting imprecise cases would be problematic.

When including events that lack fine-scale spatial details, the most granular resolution possible is usually the administrative unit level 1, which may be known as regions, departments, or states depending on the country. This loss of spatial resolution may preclude assessments of the impact of localized violence. A middle option is to exclude only the most imprecise data and aggregate remaining data to the administrative unit level 2, which may be referred to as provinces, municipalities, counties, or territories in different countries. When choosing among these spatial options, researchers must consider whether it is more important to keep all events or to measure localized exposures to armed conflict.

In the existing literature, some have handled the problem of imprecise locations by taking either of the two approaches outlined above—either eliminating the conflict events that lack a precise location or aggregating more broadly (Luetke et al. 2024; Svallfors 2022a). Yet, many do not fully describe how conflict data was processed—perhaps due to limited awareness about the peculiarities of the datasets—thereby leaving the reader unable to replicate or assess the appropriateness of their approach. In sum, the varying spatial indicators available in conflict and health datasets can present notable challenges to researchers, requiring careful and thoughtful decision-making. Approaches to these data challenges should be explicitly outlined in published materials to guide future research in the field, as well as enable replicability and comparability between studies.

##### How to address temporal imprecision.

Reports of conflict events in existing datasets may also lack exact temporal information. In Nigeria in 2018, the majority of armed conflict events reported in the UCDP data had an exact date (>80 percent), while only a tiny proportion had a temporal imprecision of longer than one month (0.2 percent) (see Table 4). Because aggregating events at the monthly level is likely appropriate for most analyses, a simple solution is to eliminate events that lack such temporal detail. Nevertheless, researchers should review the distribution of events across the temporal precision categories before moving forward. Additionally, it is important to report the number of events removed at any step of aggregation.

There are also other notable issues with the reported timing of conflict events and how events are counted in the researcher’s analysis. Since ongoing events may show up only once in the data but last several weeks, one solution is to aggregate events to a month or year. However, researchers must be careful not to count ongoing events multiple times. It is also important to describe whether and how events count in multiple aggregation categories. Another issue specific to the UCDP data is that because a conflict must be associated with 25 casualties before being included in the data, the conflict will not be counted if these 25 deaths occur across calendar years (e.g., between December 15 to January 15) (Croicu and Sundberg 2016).

The timing of health data collection could also be an issue. These data may have precise temporal information (i.e., date of interview) but are often collected with large temporal gaps in between. For example, DHS are generally conducted every 5 years but use retrospective calendars to gather some information, such as birth histories, which will enable a complete time series in countries where the survey has been consistently implemented. However, not all indicators of interest are available in this module (such as how fertility preferences may vary over time) (Svallfors 2022b).

#### Measuring and operationalizing exposure to armed conflict

In addition to the data processing and aggregation issues discussed above, researchers must determine the best way to quantify conflict exposure based on the chosen research questions. Existing studies have operationalized the spatiotemporal dimensions of conflict exposure in a multitude of ways (Grace et al. 2022).

When combining conflict data and health data, researchers often link datasets at different spatial scales (e.g., community-level conflict with individual-level health). While certain approaches result in misclassification (e.g., using the GPS points for all armed conflict events while ignoring the spatial precision indicator), there are multiple ways to appropriately measure and aggregate conflict data. While the spatial dimensions of existing conflict and health datasets are often outside of the researcher’s control, there are spatial considerations that may be easier to address.

One such consideration is the distance at which people’s daily lives are affected by armed conflict. This would have methodological implications for the inclusion area for conflict events around a particular community. For example, the decision of the inclusion distance (i.e., a buffer of a particular size) around a particular community for which there is health data should be based on contextual features of the particular geographic region under study. Alternatively, distance may matter less than connectedness and, therefore, a researcher may choose to consider the relative connectedness or isolation of a particular community, such as by incorporating spatial lags. Since different datasets offer different possibilities and due to a lack of standard measurements, researchers have used a wide range of measures of conflict exposure (Adhvaryu et al. 2021; Brown et al. 2020; Grace et al. 2021; Koren 2018). Though not inherently problematic, since different research objectives may result in different methodological approaches, studies are therefore often incomparable, preventing more generalizable conclusions of the health effects of armed conflict.

For examples of spatial dimensions of conflict measurement, Adhvaryu et al. (2021) used and compared ACLED and UCDP data, assigning the conflict incidence exposure at the spatial level of each 0.5° by 0.5° grid cell to analyze the impact of natural resources (absolute and relative to neighboring countries) on the likelihood of armed conflict (Adhvaryu et al. 2021). In a multicountry study of early-age exposure to armed conflict and exposure to intimate partner violence based on the DHS and UCDP-GED, Torrisi (2023) measured conflict exposure as households located within 15 km buffer zones centered around the coordinates of events in three out of four studied post-Soviet countries (Armenia, Moldova, and Tajikistan). In the fourth study country (Azerbaijan), exposure was defined as whether the respondent’s origin district was located in a conflict-affected region because the DHS was not geocoded in this country. Moreover, in her work on abortion and miscarriages during the Tajikistani civil war, also using the DHS and UCDP-GED, O’Brien (2020, 10) opted for measuring exposure at the more aggregate district level (administrative unit level 2) instead of more precise or aggregate units for “the best tradeoff between precision and comparability.” Further, O’Brien employed a spatial lag operator to account for the diffusion and nonrandom geographic distribution of conflict events, thereby accounting for events in neighboring districts (that were not separated by a mountain range). Events that could not be categorized at more fine-grained levels than the first administrative unit were included in the spatial lag as “neighboring events.”

Researchers also must decide how to operationalize the timing of conflict exposure, both in terms of potential time lags between exposure and outcome and variation in health responses by the duration of exposure. Possible lag times may be quite different depending on the outcome, with some impacts being immediately observable while others may occur sometime after the event. More immediate effects might include psychological trauma and stress, while more delayed effects might include increased maternal or child mortality due to limited healthcare access, increased transmission of infectious diseases, or malnutrition due to food insecurity.

The choice of how to match conflict and health data temporally also ultimately depends on the research question and the process under study, and researchers have taken different approaches to this problem. In cross-sectional analyses of health conditions and behaviors at the time of the interview—such as contraceptive use (Svallfors and Billingsley 2019) or fertility preferences (Thiede et al. 2020)—researchers may assign conflict exposure by matching events monthly for relevant durations of time leading up to the month of the interview. Additionally, many studies of pregnancy outcomes and infant health as an outcome assign exposure during different trimesters and/or the full pregnancy (Luetke et al. 2024; Oskorouchi 2019). When the outcome under study is a health event that can be pinpointed in time—such as the first time women use contraception (Williams et al. 2012) or timing of childbirth (Østby et al. 2018)—assignment to exposure is typically done in relation to that event. Other times, researchers may define exposure periods that coincide with theoretical mediators, such as when examining the role of agricultural production in studying health outcomes related to food insecurity (Grace et al. 2023; Luetke, Grace, and Gunther 2023). Armed conflict may impact agricultural production in a variety of ways, leading to hunger and other adverse health outcomes. To maximize precision, researchers should assign exposure to the finest-scale unit that makes sense for their study. In practice, this often means conflict should be aggregated at a monthly basis, with the researcher then creating lagged indicators depending on the relevant time frames for the topic under study, as opposed to relying on broader, less precise temporal aggregates such as yearly estimates. Nevertheless, the temporal precision of the conflict data in each case will determine what is possible, as discussed above.

Moreover, it remains unclear whether counts of casualties or events are more relevant for health outcomes, and what the functional form of the relationship looks like. In all likelihood, it depends on the specific health process under study. Many have opted for a continuous conflict event measure while recognizing that severity and duration may have an important and less explored impact. Some have used transformed count indicators of events or casualties to account for skewed data distributions (Leone et al. 2019; Price and Bohara 2013). Others have used categorical variables distinguishing between levels of conflict exposure intensity, such as by using median cutoffs to define low versus high levels of exposure among exposed respondents (Svallfors 2022b). Certain studies have relied on difference-in-difference techniques, conducting pre-post analyses of affected and non-affected areas (Ekhator-Mobayode et al. 2022; Torrisi 2024).

Creating and comparing different measurements of conflict exposure is important for evaluating whether there are different dimensions of conflict that are more salient for predicting health outcomes than others. For example, UCDP-GED and ACLED data both enable comparisons between different proximities, intensities, durations, and types of conflict. However, it is unusual in the literature to report on comparisons between different conflict exposure measurements (Svallfors 2025). Further, any quantitative analysis using geospatial measures of conflict exposure will be limited in the extent to which it reflects personal experiences or perceptions of violence. Measures of perceived security (Svallfors et al. 2024a) and qualitative data of lived experiences may be better suited to capture how people think about and react to a situation of increasing insecurity in their vicinity.

In the following, we draw on these multiple considerations in an empirical example of the associations between exposure to armed conflict and current contraceptive use.

### An empirical example: Armed conflict and current contraceptive use in Nigeria

To demonstrate some of the issues of using armed conflict data in health research discussed above, we provide an empirical example based in Nigeria, a country that has experienced repeated cycles of armed conflict in the past several decades with notable consequences for population health (Bendavid et al. 2021; Checchi et al. 2023; Ekhator-Mobayode et al. 2022; Svallfors et al. 2024a). Specifically, we assess the impact of armed conflict on women’s current contraceptive use.

Prior research on how armed conflict affects contraception reveals complex and sometimes contradictory effects. While Williams et al. (2012) found increased first-time contraceptive use during Nepal’s civil war, Svallfors and Billingsley (2019) documented reduced contraceptive use in Colombia, with fertility demand explaining only a small portion of this relationship. Torrisi (2024) similarly found that Mali’s 2012 insurrection was associated with decreased modern contraceptive use, particularly of shorter acting methods. These findings highlight distinct mechanisms through which conflict affects contraception: changes in fertility preferences (Svallfors 2022b; Thiede et al. 2020), disrupted access to contraceptive services due to damaged healthcare infrastructure (Bendavid et al. 2021; Levy and Sidel 2016), reduced knowledge about sources of contraception (Torrisi 2024), and undermined reproductive autonomy (Svallfors 2024). To this body of literature, our empirical example adds knowledge about contraceptive use in Nigeria, a previously understudied context in terms of sexual and reproductive health outcomes of armed conflict.

We define exposure to conflict as battle events taking place near the respondent’s area of residence, while comparing various operationalization decisions, using data from UCDP-GED and IPUMS DHS (Boyle, King, and Sobek 2022; Croicu and Sundberg 2016; National Population Commission - NPC and ICF 2019). We demonstrate several different methods of spatially and temporally linking health with conflict data and discuss the motivations for each approach.

## Methods

In this analysis, we combine three different datasets: population health data from the IPUMS DHS, armed conflict event data from the UCDP, and a shapefile for the administrative boundaries of Nigeria. All three datasets are linked spatially, and the conflict and health data are also linked temporally, as described below.

### Armed conflict event data.

We employed conflict data from the latest version of the UCDP-GED (version 22.1) for Nigeria (Croicu and Sundberg, 2016). We included all conflict events in Nigeria between August 1, 2017, and December 31, 2018, to provide a full year of data before the 2018 DHS in Nigeria. We created three different measurements of conflict based on the date of the DHS interview and the spatiotemporal information provided for each conflict event. Further details of UCDP-GED data are provided above.

### Population health data.

We use IPUMS DHS because it is a cleaned, harmonized, and well-documented version of the raw DHS data that allows for comparison across countries. We use data from the 2018 round of the DHS in Nigeria, which were collected between August 14, 2018, and December 29, 2018, and sampled women aged 15–49 (National Population Commission - NPC and ICF 2019). DHS includes information about women’s contraceptive use at the time of the interview and a variety of sociodemographic characteristics, as well as geographic reference points that enable spatial linking between the surveys and conflict data.

Of the 1,389 DHS clusters in the 2018 Nigerian DHS, only 1,382 had GPS coordinates. To enable geolocated data linkage, we removed seven clusters (*n* = 198) without geospatial data. As our outcome was contraceptive use, we further restricted our analytical sample to women who were not currently pregnant (*n* = 4,176), resulting in 37,447 respondents. Lastly, to correctly assign conflict exposure, we excluded women who were just visitors to the area (*n* = 206) and those who had lived in the area less than a year (*n* = 1,462). Our final analytic sample thus included 36,030 women aged 15–49.

### Outcome variable.

Our dependent variable was the current use of a contraceptive method. The original variable was categorical and distinguished between no method, folkloric method, traditional method, and modern method. For our purposes, we collapsed all the methods into a binary variable measuring whether or not the respondent was using any contraception.

### Covariates.

Following existing research in this field (Luetke et al. 2024; Svallfors and Billingsley 2019; Torrisi 2024), we included the following respondent characteristics as potential confounders of the relationship between armed conflict and contraceptive use: age (in 5-year categories), partnership status, educational attainment, socioeconomic status (in the form of wealth quintiles), urban or rural residence, number of children ever born (i.e., parity), and age of youngest child if the respondent reported having living children.

### Data cleaning and harmonization steps.

In addition to more basic data cleaning and recoding of variables, we also conducted several steps to harmonize our data before merging. To match the shapefile of Nigeria and the UCDP-GED data for aggregation to the first or second administrative level, we compared the columns including the administrative boundary names. In this process, we conducted several natural language processing steps to remove any discrepancies between the names of each administrative unit value in order to later merge datasets based on these columns. This included removing accents and dashes; making the capitalization consistent across datasets; and correcting any spelling differences in area names. Where we could not find a close match (i.e., administrative boundary names were mislabeled and unable to be manually matched) or the value was completely missing, we conducted a spatial intersection using the geolocation of the armed conflict data and the shapefile for Nigeria in order to identify the corresponding administrative boundary names. We also conducted the same cleaning process on the DHS dataset for administrative units 1 and 2 before linking data. Since administrative subdivisions in Nigeria all have unique names, this was a relatively straightforward process compared to settings where multiple units may share the same name.

To prepare for temporal matching, we transformed the temporal data in both sets. In the conflict data, we transformed the date of each event into century-month codes (CMCs). We then summed the number of conflict events by month and year. DHS data report the interview date for each respondent, which we also transformed into CMC. Since we aggregated to the month level, we excluded events that lacked monthly temporal precision (only 0.2 percent of the events) (see Table 4).

### Creating the exposure measure: Spatial and temporal aggregation steps.

The DHS and the UCDP-GED both have spatial and temporal values. To join these two datasets spatiotemporally and to demonstrate how different choices impact the analysis, we linked the data in several ways based on various levels of spatial and temporal precision.

For the first level of precision, where the exact location of the conflict event is known, we created 2 km circular buffers around GPS points in urban areas and 5 km buffers around GPS points in rural areas in alignment with the displacement protocol used by the DHS. This approach guarantees that our buffers contain the true centroid for at least 99 percent of all enumeration areas. Next, we eliminated UCDP-GED events lacking an exact location (see Table 3). Lastly, we summed the number of events at the DHS cluster level by conducting a spatial intersection to see how many conflict events occurred within the DHS buffer zones. This resulted in an indicator of conflict exposure aggregated at the DHS cluster level. This is a very conservative or localized measure of armed conflict, as we are demonstrating the narrowest buffer area that is used in research evaluating contextual or environmental exposures. A broader buffer area may be appropriate if it is plausible that armed conflict occurring nearby, but not as close as these smaller buffer areas, would impact the outcome under investigation in the specific study.

For the second level of spatial precision, where the location of the conflict event is known within a 25-km radius, we used the same buffering process for the DHS clusters as described above (for precision level 1). Here, we eliminated less precise events and created 25 km buffers around each geolocated conflict event. Next, we added back events that had more precise spatial data (i.e., at precision 1, where the exact location was known and reported). Finally, we conducted a spatial intersection, capturing all events at precision 1 that fell into the DHS cluster buffer and all events at precision level 2 where the 25-km buffer overlapped with the DHS cluster buffer area. Again, this resulted in an indicator of conflict exposure summarizing the number of events at the DHS cluster level, but this time including events for which the location was known to be within 25 km of the reported location (i.e., precision level 2), in addition to events with an exact GPS location (i.e., precision level 1).

For the third level of precision, where the location of the conflict event is known within an administrative unit at level 2 (in Nigeria, 774 LGAs), we summarized events with records of the exact location, the location within 25 km, and the location at the administrative unit level 2.

For the fourth and highest level of aggregation, where the location of the event is known only at the administrative unit level 1, in Nigeria referred to as states and one territory (the Federal Capital Territory of Abuja and 36 states), we included UCDP data that had the exact location, the location within 25 km, at the administrative unit level 2, and the administrative unit level 1.

For all levels of spatial aggregation, we created three separate conflict exposures that summarized conflict events across the duration of exposure before the interview month: (1) the prior 3 months, (2) the prior 6 months, and (3) the prior 12 months. This resulted in an individualized exposure for each respondent across four spatial aggregations and three temporal aggregations.

### Data operationalization.

To assess how differences in the operationalization of conflict affect results, we created three measures of conflict. For the first, we operationalized conflict exposure as continuous variables summarizing the number of events over the three durations (3, 6, and 12 months) in the area where the respondents resided. Next, because the continuous variable was heavily skewed toward zeros (i.e., zero-inflated), we also created binary armed conflict exposures where 0 represented no conflict exposure (i.e., no events) and 1 represented conflict exposure (i.e., 1 or more events) for the same spatiotemporal specifications. Lastly, to identify potential dose–response effects and account for the high share of unexposed respondents, we also categorized continuous indicators into levels of exposure, distinguishing between whether the respondent was exposed to, for example, no, low, or high levels of conflict by splitting exposed respondents into a low- or high-exposure group according to the 50th percentile observed in the sample (i.e., at the median). While beyond the scope of our empirical exercise, alternative operationalization might have been to use zero-inflated transformed continuous indicators.

### Statistical analysis.

To explore the relationship between armed conflict and current contraceptive use, we employed a series of linear probability models with fixed effects at the level one step coarser than the level at which the exposure was measured. For example, where the armed conflict exposure was measured at the DHS cluster level (as is the case for the conflict measures at Precision 1 and Precision 2), the fixed effect is the administrative level 2. At the coarsest level (i.e., Precision 4), there is no coarser spatial level and, therefore, the fixed effects are removed for those models. While matching the fixed-effects levels to the exposure level would have been advisable, this was not possible here due to collinearity issues. All models have cluster-adjusted standard errors at the DHS cluster level to account for the similarities between women residing in the same communities. We then generated forest plots to present the point estimates for the relationship between armed conflict—measured over three different time periods (past 3, 6, and 12 months) and at the varying spatial precision levels—and current contraceptive use.

## Findings

Descriptive statistics of the sampled population are presented in Online Appendix 1. Overall, 15 percent of sampled women reported using any contraceptive method at the time of the survey. Below, we present results from three sets of models that operationalize armed conflict exposure using continuous, binary, and categorical measures of armed conflict. All results shown are from fully adjusted models; models adjusted only for respondents’ age are presented in the Online Appendix (see Figures A2–A4) and yield very similar estimates.

### Continuous measure of armed conflict.

We first examine the associations between current contraceptive use and continuous measures of conflict exposure across three exposure windows (past 3, 6, and 12 months) and four spatial precision levels (Figure 2). Across most model specifications, point estimates are close to the null value of zero and the confidence intervals include the null, indicating limited evidence of an association between armed conflict and current contraceptive use.

However, three specifications reveal statistically significant associations between armed conflict and current contraceptive use. When the conflict exposure is measured at the finest spatial resolution (Precision Level 1) and within the prior 3 months, estimates indicate a small but statistically significant negative association, where exposure to one additional conflict event is associated with a 0.2 percentage point decrease in the probability of current contraceptive use. This association attenuates and becomes statistically insignificant in the model specifications where the conflict exposure is measured over longer temporal windows (i.e., over 6 and 12 months).

The other two significant estimates are observed at the 3-month and 12-month temporal specifications and at Precision Level 3. The estimates of greatest magnitude are observed in the 3-month window specification, where one additional conflict event is associated with a 1.1 percentage point increase in the probability of current contraceptive use. Meanwhile, the other significant association is observed in the 12-month window specification, but the estimate is smaller in magnitude (a 0.2 percentage point increase) and only marginally significant compared to the 3-month specification.

Estimates for Precision Level 2 and Precision Level 4 are tightly centered around zero with narrow confidence intervals and do not provide evidence of a statistically meaningful association. Overall, we do not observe a consistent pattern in significance or effect size across temporal windows or spatial precision scales.

### Binary measure of armed conflict.

Next, we examine the effect of a *binary* indicator of armed conflict exposure across the same spatial and temporal specifications (Figure 3). As with the continuous exposure models, most estimates cluster around the null value of zero, indicating little evidence of a relationship between conflict exposure and current contraceptive use.

However, we do again observe a significant negative association between armed conflict and current contraceptive use at the Precision Level 1 and 3-month model specification, where being exposed to any conflict is associated with a 6.4 percentage point decrease in the probability of current contraceptive use compared to not being exposed to any conflict. Again, we see that the significance of this association dissolves for the same precision level but at broader temporal measures of armed conflict (i.e., the 6-month and 12-month windows).

In contrast, we observe statistically significant positive associations only for the models in which conflict exposure is measured at the broadest spatial resolution (Precision Level 4) and across all three temporal windows. In these models, exposure to any violence is associated with a 3.6–4.3 percentage point increase in the probability of current contraceptive use when compared to no exposure to armed conflict.

As with the continuous models, results vary by spatial and temporal specifications, though the negative association observed at the most precise and temporally narrow specification mirrors findings from the continuous exposure models.

### Categorical measure of armed conflict.

Finally, we examine the effect of a *categorical* measure of conflict exposure on current contraceptive use (Figure 4), comparing respondents exposed to low and high levels of conflict to the unexposed across the same spatial and temporal specifications. Again, many estimates are nonsignificant and close to the null. However, there are several specifications that reveal significant associations.

At the finest spatial resolution (Precision Level 1), higher levels of conflict exposure are associated with a statistically significant decrease in the probability of current contraceptive use for the 3- and 6-month exposure windows compared to no exposure to conflict (a 7.7 percentage point and 7.5 percentage point decrease, respectively), but not at the 12-month exposure window. For these same model specifications, estimates for lower levels of conflict exposure vary in direction and significance, sometimes indicating a modest increase and sometimes a modest decrease in contraceptive use relative to no exposure.

At the coarsest spatial resolution (Precision Level 4), both low and high levels of conflict exposure are consistently associated with statistically significant increases in the probability of contraceptive use across all temporal windows when compared to no exposure to conflict. Low levels of exposure are associated with increases—ranging from 3.7 to 5.5 percentage points—in the probability of contraceptive use, while high levels of exposure are associated with smaller but still significant increases—ranging from 1.9 to 3.5 percentage points—in the probability of contraceptive use. Rather than exhibiting a clear dose–response relationship, these results reveal divergent patterns across spatial scales, with negative or null associations at the most precise spatial resolution level (Precision 1 models) and more consistent positive associations at the broadest spatial resolution level (Precision 4 models). For the spatial precision levels in between, we observe that estimates are mostly insignificant and clustered around the null value of zero.

## Discussion

In the current geopolitical context of multiple ongoing armed conflicts, the lives and health of individuals and communities continue to be profoundly affected (Hedström and Herder 2023; Iqbal 2010; Wise 2017). Research can make significant contributions to understanding and addressing these impacts. As such, the rigor and validity of research are paramount, both to enable replicability and to produce accurate information to guide policy and interventions aiming to reduce the human costs of armed conflicts. Researchers studying the effects of armed conflict on health outcomes must carefully consider several notable methodological choices, including dataset selection, spatial and temporal aggregation, and ways of operationalizing armed conflict exposures. This study highlights best practices for navigating these complexities and provides guidance on conducting rigorous and replicable research on armed conflict and population health outcomes. Specifically, we focused on the structure of commonly used armed conflict datasets, potential sources of bias, and aggregation and analytic decisions.

Through an empirical example analysis, we demonstrate how methodological and analytical decisions can influence the associations observed from statistical models. For example, in our case study of Nigeria, we show that the estimates of the association between conflict exposure and current contraceptive use vary in both magnitude, direction, and significance depending on whether conflict is operationalized as continuous, binary, or categorical, and the spatial and temporal aggregation choices. Our findings reveal some trends, including generally significant negative associations between conflict and contraceptive use at the finest temporal and spatial scales (i.e., Precision Level 1 and 3-month temporal window) and across continuous, binary, and categorical operationalizations. In contrast, we observe almost the exact opposite trend for the coarsest spatial aggregation across all temporal windows (i.e., Precision Level 1 and 3-, 6-, and 12-month temporal windows) but only for the binary and categorical operationalizations of the armed conflict exposure. These results underscore the importance of considering spatial and temporal dimensions of exposure both when designing studies and interpreting study findings.

This study also advances substantive knowledge by investigating the association between armed conflict and current contraceptive use in the context of Nigeria, a context that has received limited attention regarding the population health consequences of conflict. Our divergent findings suggest that finer-scale spatial and temporal aggregations may better capture more immediate and proximate disruptions to healthcare access and services, thus resulting in a reduced probability of contraceptive use in these models. For example, prior research demonstrates that exposure to localized armed conflict reduces access to healthcare services such as prenatal care (Chukwuma and Ekhator-Mobayode 2019) and childhood vaccination (Sato 2019) as well as increases in intimate partner violence (Ekhator-Mobayode et al. 2022), which could explain our estimates showing lower contraceptive use in areas affected by nearby armed conflict.

In contrast, coarser aggregations may dilute the effects of armed conflict, attenuating negative impacts or even producing apparently positive associations. This could be because of ecological fallacy or because the armed conflict exposure is averaged across numerous, heterogeneous areas and communities. It may also be that some health behaviors and outcomes may still be influenced by broader level armed conflict. Some existing research supports this, indicating that conflict-affected men and women in Nigeria are more permissive of contraception, abortion, and early marriage as a response to security concerns and armed conflict exposure, all of which may be interpreted as risk-averse responses to war (Svallfors et al. 2024a). This may explain the higher probability of contraceptive use observed in the broader spatial aggregation models, since such behaviors are likely influenced even if the armed conflict is known to the community but not necessarily happening within the community.

However, considering the sensitivity to spatial and temporal dimensions of conflict exposure, more research is needed to ascertain the processes through which exposure to armed conflict affects reproductive autonomy, healthcare access, fertility preferences, and other mechanisms that may influence contraceptive use. Future research may also investigate effect moderation across intersectional social groups to ascertain whether conflict interacts with social inequality in shaping contraceptive use and other population dynamics (Bauer 2014). Additionally, future research should be grounded in qualitative data collection to capture the context-specific and heterogeneous experiences of women within particular family units and communities.

Establishing causal relationships between armed conflict exposure and health outcomes is critical for informing public health policy and interventions. At the same time, the unique methodological challenges inherent to conflict settings—including data availability, nonrandom assignment to treatment, and potential self-selection out of treatment—require careful consideration when selecting and implementing causal inference approaches. More specifically, randomized experiments are neither feasible nor ethical for exposures such as armed conflict, and observational methods that approximate or mimic randomization often rely on strong, untestable assumptions about study populations. In addition, the mismatch between the high-frequency timing of conflict event data (often available at the daily level) and the relatively sparse collection of health survey data (often only available every 3–5 years) limits the ability to establish the necessary temporal ordering between exposure and outcome. Lastly, for complex exposures like armed conflict, exposures may interact with multiple factors at varying levels; thus, causal inference methods may oversimplify relationships in ways that do not reflect reality (Turner et al. 2025; van der Maat 2021).

Observed differences between studies of the same outcome (e.g., contraceptive use) may result from differences in methodological strategies (e.g., analytical method or sample inclusion criteria) or from genuine differences in the effects of conflict on health across contexts. While our goal is not to suggest a standard way of conducting research on conflict and population health, it is important that researchers maintain transparency in coding and data decisions to facilitate evaluations of comparability between different methodologies and findings. Presenting precise data reduction protocols in an Online Appendix or sharing data management code are two strategies that could increase such transparency.

The variability in both magnitude and direction of the point estimates across our model specifications illustrates how different choices in spatial and temporal aggregation can meaningfully shape analytic conclusions. Therefore, aggregation choices, alongside other methodological decisions, should be considered carefully, explicitly described in this paper, and justified by a logical rationale. Each matching strategy will have implications for the results and should ultimately be selected based on the research question and theorized mechanisms. For example, while geocoded analyses of nearby violence in a 20-km buffer may offer greater precision and relevance for pathways such as stress or healthcare service disruption while aggregating at administrative subdivisions might be relevant for capturing behavioral changes in contraceptive use that may be influenced by implied hearing about nearby armed conflict. Further, it is important to consider the appropriate duration of exposure, as the time between exposure and effect on the outcome might differ. For example, when the outcome under study is infant health, the relevant window of exposure might be limited to the pregnancy and possibly before conception, while the effect of conflict on mental health outcomes may accumulate with longer exposure times. Since very few studies have compared geocoded to subdivision approaches to different temporal dimensions, it is still an open question of how to best operationalize exposure to armed conflict. Most importantly, researchers should be transparent about their choices and perform sensitivity analyses of how results vary depending on which spatial and temporal approach is used. Further, in order to accurately interpret and understand the mechanisms linking exposure to armed conflict with health outcomes, it is important to collect qualitative data and use such data to illuminate and explain empirical quantitative findings and statistical associations.

In addition to the study-specific choices, certain issues are relevant to all studies employing such datasets, such as the spatiotemporal precision of events. Whether or not existing datasets represent a complete picture of armed conflict, they provide the most comprehensive information available and—when used properly—can significantly advance our understanding of how armed conflict impacts population health and other outcomes. In research on population health in armed conflict, the issues of spatiotemporal granularity and precision, measurement, and how these factors shape the lived experiences of people in conflict settings are salient topics to be considered but remain relatively unexplored in the existing literature. Given the increasing attention from researchers on the deleterious consequences of conflict on population health, this study advances methodological discussions in the field (Axinn, Ghimire, and Williams 2012; Moreno-Serra et al. 2022; Wise 2017). Our study can guide researchers to make informed, deliberate decisions, producing more rigorous, transparent, robust, and replicable findings.

Although this study highlighted quantitative data and methods in armed conflict and health research, exposure to armed conflict—and violence more broadly—is deeply personal with wide-ranging impacts that vary across cultures, communities, and individuals. As such, people residing in the same community and exposed to similar levels of violence may experience and respond very differently. Because this exposure is complex and heterogeneous in its impacts, we encourage the collection of qualitative data around individual experiences, perceptions, and consequences of armed conflict either on its own or to supplement quantitative analyses. Quantitative data are useful for revealing population-level trends and the “big picture” of an exposure or phenomenon, but they rarely capture the specifics of individual experiences or how these may differ from person to person.

In future research, we encourage mixed-methods approaches that integrate qualitative and quantitative data in order to both estimate overall relationships between armed conflict and health outcomes and capture the particularities of how these relationships affect individuals. Mixed-methods and qualitative studies have particular strengths in revealing patterns and pathways between exposures and outcomes that may go unrecognized or unobserved in quantitative studies. Further, the lived experiences of people affected by conflict can inform the conceptualization and operationalization of meaningful exposure measures. Innovative methods—including adding specific modules that query individual-level conflict exposure and impact to large-scale population health surveys, especially in conflict-affected settings—should be used to better incorporate the experiences of people exposed to armed conflict. For example, the World Values Survey asks about perceptions of (neighborhood) security, which could be aggregated at different levels to capture how respondents perceive the security situation in their area of residence (Svallfors et al. 2024b). The Vietnamese Health and Aging Study asks detailed questions about experiences of wartime stressors, such as forced displacement and food/water insecurity, in addition to including geospatial measures of aerial bombings (Korinek et al. 2019). Combining spatiotemporal and perception measures can enhance the nuance and validity of studies. Finally, qualitative methods add the richness of personal experience, helping interpret, contextualize, and inform policy recommendations from study findings.

## Conclusions

Publicly available subnational data on events of armed conflict, combined with representative health data, now allow for a nuanced assessment of how exposure to conflict affects population health. However, researchers face numerous dilemmas when using such data, including their varied temporal and spatial precision, which carries important methodological implications. With growing attention to the impact of conflict on population health, there is a risk that these datasets will be used without consideration of potential issues, leading to spurious or biased results. We recommend some concrete strategies for the responsible use of such data in public health research. Moreover, we emphasize the importance of transparency and deliberate decision-making in this emerging field, ensuring that future research will build a body of reliable and comparable evidence on the impacts of armed conflict on population health.

## Supplementary Material

Supplemental Material

## Figures and Tables

**FIGURE 1:**
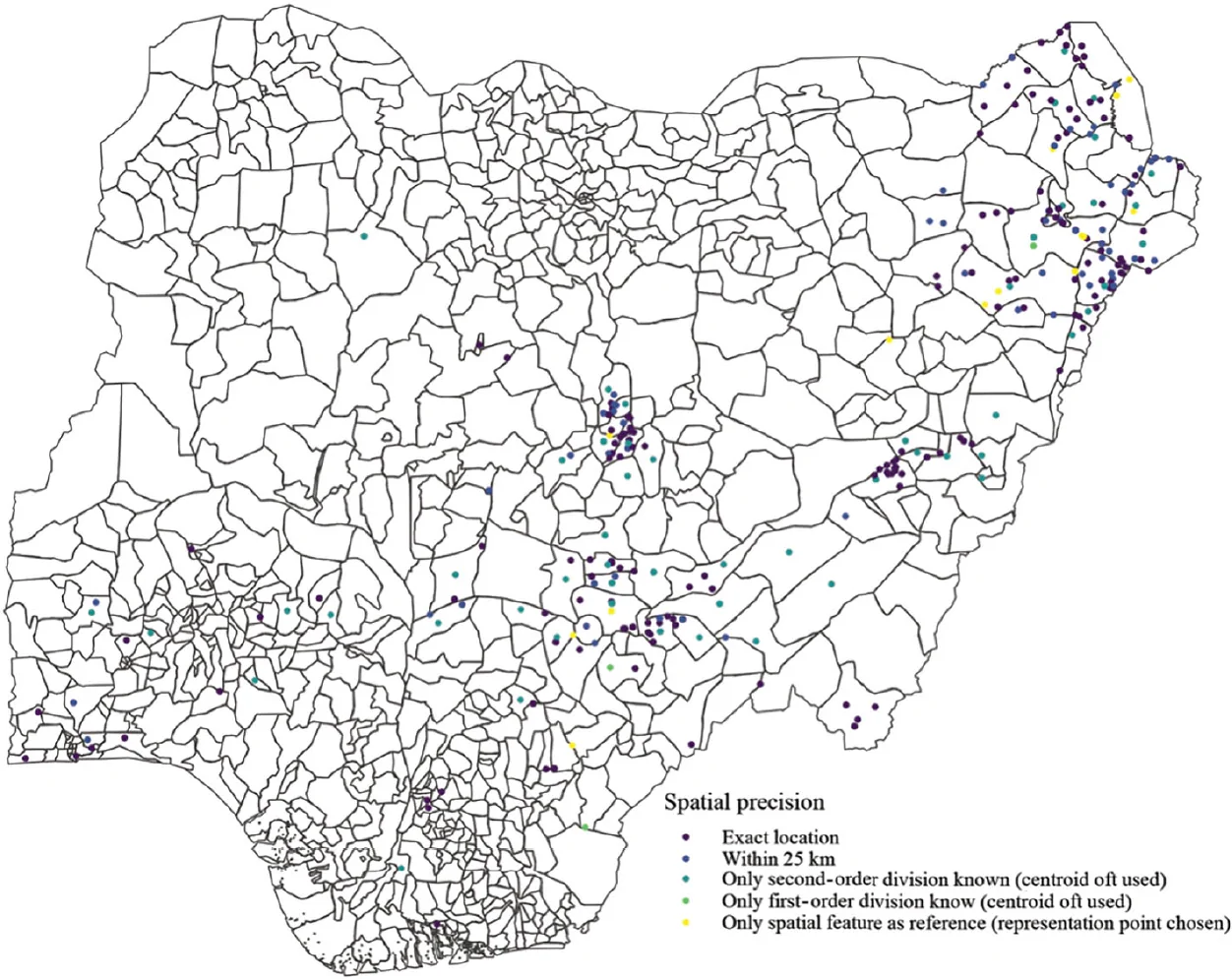
Reported armed conflict events in Nigeria in 2018, by level of spatial precision SOURCE: UCDP-GED dataset.

**FIGURE 2:**
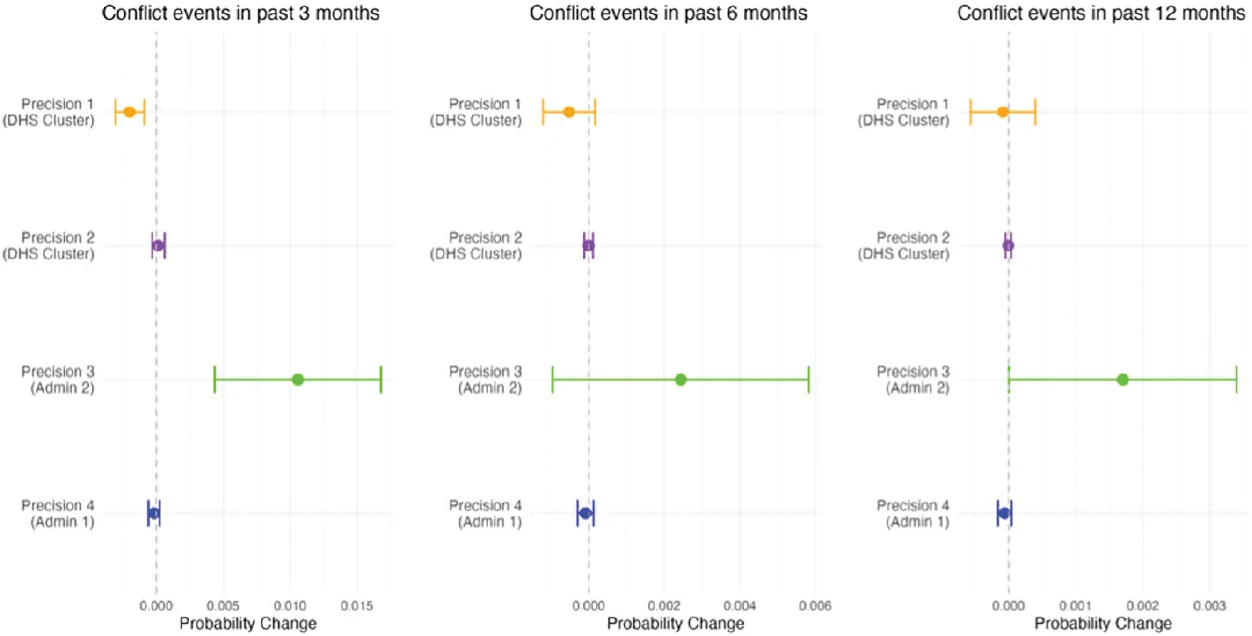
Forest plot of models of the association between continuous armed conflict exposure and current contraceptive use, fully adjusted NOTE: These estimates are drawn from linear probability models with fixed effects at the level one step coarser than the level at which the exposure was measured. For example, at Precision 1 and 2, where the armed conflict exposure was measured at the DHS cluster level, the fixed effect is the administrative level 2. At the coarsest level (i.e., Precision 4), there is no coarser spatial level and, therefore, the fixed effects are removed. All models have cluster-adjusted standard errors at the DHS cluster level. These models are adjusted for the age of the respondent (in 5-year categories), partnership status, educational attainment, socioeconomic status (in the form of wealth quintiles), urban or rural residence, number of children ever born (i.e., parity), and age of youngest child if the respondent reported having living children.

**FIGURE 3:**
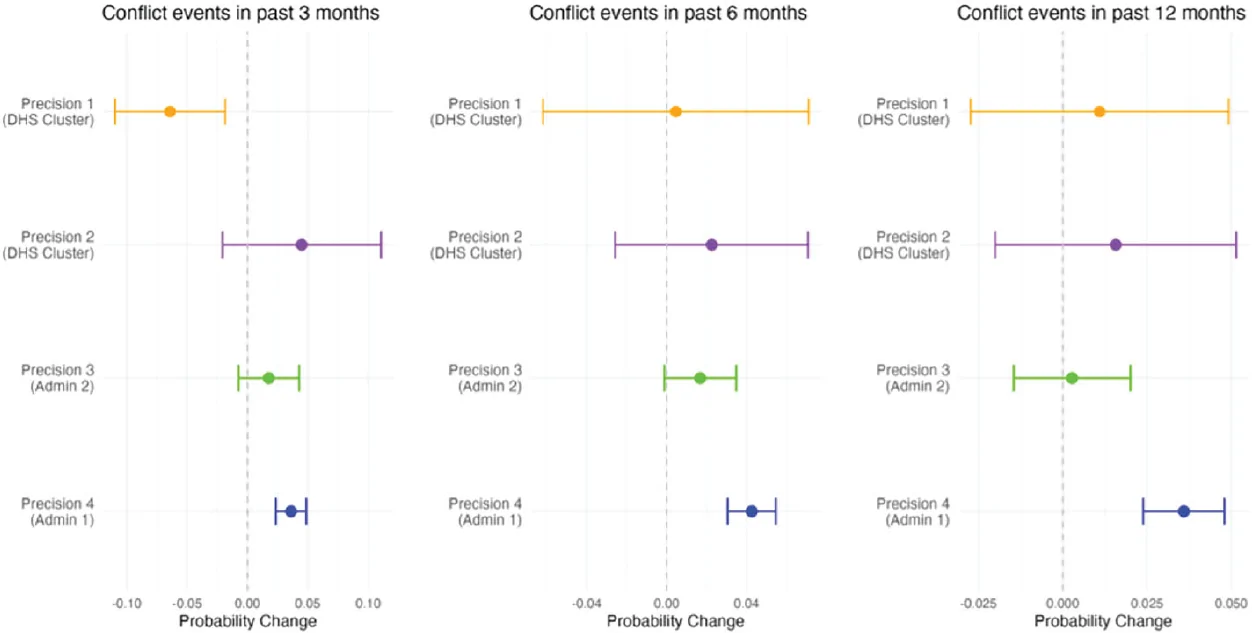
Forest plot of models of the association between binary armed conflict exposure and current contraceptive use, fully adjusted NOTE: These estimates are drawn from linear probability models with fixed effects at the level one step coarser than the level at which the exposure was measured. For example, at Precision 1 and 2, where the armed conflict exposure was measured at the DHS cluster level, the fixed effect is the administrative level 2. At the coarsest level (i.e., Precision 4), there is no coarser spatial level and, therefore, the fixed effects are removed. All models have cluster-adjusted standard errors at the DHS cluster level. These models are adjusted for the age of the respondent (in 5-year categories), partnership status, educational attainment, socioeconomic status (in the form of wealth quintiles), urban or rural residence, number of children ever born (i.e., parity), and age of youngest child if the respondent reported having living children.

**FIGURE 4:**
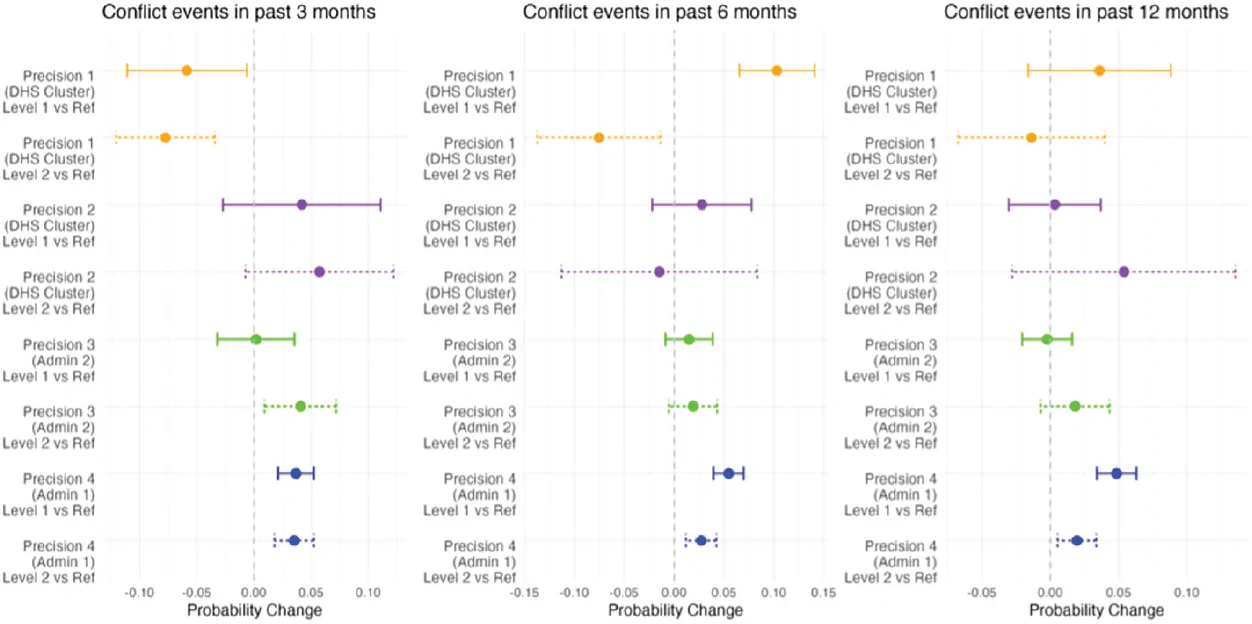
Forest plot of models of the association between categorical armed conflict exposure and current contraceptive use, fully adjusted NOTE: These estimates are drawn from linear probability models with fixed effects at the level one step coarser than the level at which the exposure was measured. For example, at Precision 1 and 2, where the armed conflict exposure was measured at the DHS cluster level, the fixed effect is the administrative level 2. At the coarsest level (i.e., Precision 4), there is no coarser spatial level and, therefore, the fixed effects are removed. All models have cluster-adjusted standard errors at the DHS cluster level. These models are adjusted for the age of the respondent (in 5-year categories), partnership status, educational attainment, socioeconomic status (in the form of wealth quintiles), urban or rural residence, number of children ever born (i.e., parity), and age of youngest child if the respondent reported having living children.

**TABLE 1:** Similarities and differences between UCDP-GED and ACLED datasets

Characteristics	UCDP-GED	ACLED

Conflict definition	Focuses on **organized violence with at least 25 fatalities** associated with a particular conflict within a given calendar year	Focuses **broadly on political violence** but also includes protests and demonstrations of more than three people
Inclusion criteria	Requires a minimum of **25 deaths associated with a particular conflict per calendar year to be included in the dataset**	No minimum thresholds for inclusion, thus, **includes both lethal and non-lethal conflict events**
Sources	Two-pass system from traditional media and then validated by reports from NGOs and other organizations, local news outlets, and others on the ground	Drawn from an amalgamation of traditional media, reports from NGOs and other organizations on the ground, local partners, and social media
Type of conflict	– State-based conflict – Non-state conflict – One-sided violence (e.g., state or non-state attacks on civilians)	– Battles – Riots and protests – Violence against civilians – Strategic developments – Explosions/remote violence
Geographic coverage	**Nearly global**, ~150+ countries	**Nearly global**, ~160+ countries
Temporal coverage	**Global coverage from 1989** (and a few specific conflicts have data available from 1981)	Coverage from 1997 on the African continent and **global coverage since 2015**
Frequency of updating	**Annually**, occasionally more frequent updates in particular cases (They update the dataset when new information is available)	**Weekly**, providing almost real-time reporting (They update the dataset when new information is available)
Use case	– Research focused on lethal violence (i.e., narrower, more focused definition of violence) – Comparability studies across time and countries for consistent and reliable coding of events over time	– Research focused on a broader scope of violent events (i.e., more than just lethal violence) – Real-time reporting and monitoring of armed conflict and violence – Early warning systems

SOURCE: Data for this table were drawn from ACLED (2024) and Croicu and Sundberg (2016).

**TABLE 2:** Potential biases in armed conflict and population health analyses

Realm of bias	Type of bias	Description	Example	Expected bias direction

Spatial	Random spatial misclassifi cation	Armed conflict events are misplaced in space but randomly, meaning not associated with exposure or outcome	Armed conflict happened in town A in one district but is assigned to town B in a different district	– Bias toward null – Can distort typical patterns of spatial correlation
Spatial	Urban spatial misclassification	Armed conflict events reported more in urban or more populated places; rural armed conflict events missed because fewer observers/witnesses	Armed conflict happened in a rural setting but is reported as having happened in a nearby urban center	– Bias toward the null – Possible overestimate of harms in urban/populated areas – Can distort typical patterns of spatial correlation
Spatial	Reporting infrastructure spatial misclassification	Spatial misreporting that occurs systematically within certain areas (e.g., regions with better reporting infrastructure)	Conflict events are more likely to be reported in districts or regions with a greater number of NGOs or larger media presence	– Bias may be toward or away from the null, depending on the outcome distribution – Can distort typical patterns of spatial correlation
Temporal	Random temporal misalignment	Armed conflict events reported on the wrong day, but this misreporting is not related to exposure or outcome	Armed conflict event occurred on September 16, 2025, but actually occurred on August 16, 2025	– Bias toward null – Temporal alignment between exposure and outcome weakened or erroneous
General	Eliminating imprecision data or data lacking important details for analysis	Dropping events with incomplete details (e.g., unknown location or date)	Events without GPS coordinates or without an exact date are excluded	Bias may be toward or away from the null depending on whether excluded events differ systematically
General	Underreporting in inaccessible or active conflict areas	Events in remote or fragile/insecure locations are less likely to be recorded	Fewer violent exchanges are reported in active, ongoing conflicts because too dangerous for reporters to be in active conflict zones	– Bias away from the null if these areas have more severe outcomes – Possible underestimate of exposure if excluding the most extreme violent events – Important for real-time reporting but data are often updated over time when additional information becomes available
General	Purposeful distortion of armed conflict data (concealment of events and deaths)	Actors/combatants deliberately under- or over-report events to support political objectives of particular actors	Governmental forces report fewer civilian deaths than actually occurred, or rebel groups exaggerate fatalities of opposing groups	– Bias may be toward or away from the null, depending on the direction of data manipulation – Possible spurious results (i.e., threatens validity of causal inference)

**TABLE 3:** Spatial precision of UCDP armed conflict events in Nigeria, 2018

Spatial precision of conflict event	*N* = 534^a^

Exact location	243 (46%)
Within 25 km	107 (20%)
Only the second-order division is known (centroid oft used)	146 (27%)
Only the first-order division is known (centroid oft used)	7 (1.3%)
Only the spatial feature as a reference (the representation point chosen)	31 (5.8%)
Only the country known	0 (0%)
Event occurred in international waters or airspace	0 (0%)

a *n* (%).

**TABLE 4:** Temporal precision of UCDP armed conflict events in Nigeria, 2018

Temporal precision of conflict event	*N* = 5341^a^

Exact date known	430 (81%)
Date known in a 2–6 day range	84 (16%)
Date known in a 7 day range	5 (0.9%)
Date known in an 8–30 day range	14 (2.6%)
Date range longer than 1 month but less than a year	1 (0.2%)

a *n* (%).
