# Supplementary material for: Measuring the Impact of Armed Conflict on Population Health: A Guide for Researchers
Source: Popul Dev Rev. Author manuscript; Available in PMC 2026 Oct 15. (PMC13662798; doi:10.1111/padr.70047)
Supplement: Supplementary file 1 [file NIHMS2186216-supplement-Supplemental_Material.docx]

**APPENDIX**

| **TABLE A1 Sociodemographic characteristic of analytic sample** | |
| --- | --- |
|  | **N = 36,030^1^** |
| ***Using a contraceptive method*** |  |
| No | 30,689 (85.2%) |
| Yes | 5,341 (14.8%) |
| ***Age of respondent (in years)*** | 29.5 (10.0) |
| ***Age of respondent (in years)*** |  |
| 15-19 | 7,550 (21.0%) |
| 20-24 | 5,548 (15.4%) |
| 25-29 | 5,723 (15.9%) |
| 30-34 | 4,959 (13.8%) |
| 35-39 | 4,722 (13.1%) |
| 40-44 | 3,768 (10.5%) |
| 45-49 | 3,760 (10.4%) |
| ***Partnership status*** |  |
| Never married | 10,037 (27.9%) |
| Married | 23,037 (63.9%) |
| Cohabiting | 840 (2.3%) |
| Widowed | 1,072 (3.0%) |
| Divorced/separated | 1,044 (2.9%) |
| ***Level of Education*** |  |
| No education | 12,204 (33.9%) |
| Primary | 5,597 (15.5%) |
| Secondary | 14,470 (40.2%) |
| Higher | 3,759 (10.4%) |
| ***Urban or Rural Residence*** |  |
| Urban | 14,722 (40.9%) |
| Rural | 21,308 (59.1%) |
| ***Wealth Quintiles*** |  |
| Quintile 1 | 6,578 (18.3%) |
| Quintile 2 | 7,130 (19.8%) |
| Quintile 3 | 7,585 (21.1%) |
| Quintile 4 | 7,689 (21.3%) |
| Quintile 5 | 7,048 (19.6%) |
| ***Total children ever born to respondent*** | 4.1 (3.0) |
| ***Age of youngest child (in years)*** |  |
| No children | 10,735 (30.1%) |
| 0-2 | 16,338 (45.8%) |
| 3-5 | 4,254 (11.9%) |
| 6+ | 4,338 (12.2%) |
| ^1^n (%); Mean (SD) | |

**FIGURE A2	Forest plot of models of the association between *continuous* armed conflict exposure and current contraceptive use, adjusted only for age**

Note: These estimates are drawn from linear probability models with fixed effects at the level one step coarser than the level at which the exposure was measured. For example, at Precision 1 and 2 where the armed conflict exposure was measured at the DHS cluster level, the fixed effect is the administrative level 2. At the coarsest level (i.e., Precision 4), there is no coarser spatial level and, therefore, the fixed effects are removed. All models have cluster-adjusted standard errors at the DHS cluster level. These models are only adjusted for the age of the respondent (in 5-year categories).

**FIGURE A3	Forest plot of models of the association between *binary* armed conflict exposure and current contraceptive use, adjusted only for age**

Note: These estimates are drawn from linear probability models with fixed effects at the level one step coarser than the level at which the exposure was measured. For example, at Precision 1 and 2 where the armed conflict exposure was measured at the DHS cluster level, the fixed effect is the administrative level 2. At the coarsest level (i.e., Precision 4), there is no coarser spatial level and, therefore, the fixed effects are removed. All models have cluster-adjusted standard errors at the DHS cluster level. These models are only adjusted for the age of the respondent (in 5-year categories).

**FIGURE A4	Forest plot of models of the association between *categorical* armed conflict exposure and current contraceptive use, adjusted only for age**

Note: These estimates are drawn from linear probability models with fixed effects at the level one step coarser than the level at which the exposure was measured. For example, at Precision 1 and 2 where the armed conflict exposure was measured at the DHS cluster level, the fixed effect is the administrative level 2. At the coarsest level (i.e., Precision 4), there is no coarser spatial level and, therefore, the fixed effects are removed. All models have cluster-adjusted standard errors at the DHS cluster level. These models are only adjusted for the age of the respondent (in 5-year categories).
